# High risk of infection in ‘real‐world’ patients receiving ibrutinib, idelalisib or venetoclax for mature B‐cell leukaemia/lymphoma

**DOI:** 10.1111/ejh.13928

**Published:** 2023-02-15

**Authors:** Amanda Tey, James Schwarer, Robert Raffa, Emily Shi, Eldho Paul, Stephen Opat, Claire Dendle, Jake Shortt

**Affiliations:** ^1^ Pharmacy Department Monash Health Clayton Victoria Australia; ^2^ Monash Infectious Diseases Monash Health Clayton Victoria Australia; ^3^ Monash Centre for Health Research and Implementation, School of Public Health and Preventive Medicine Monash University Clayton Victoria Australia; ^4^ Department of Medicine, School of Clinical Sciences at Monash Health Monash University Clayton Victoria Australia; ^5^ Monash Haematology Monash Health Clayton Victoria Australia

**Keywords:** ibrutinib, idelalisib, infection, lymphoma, venetoclax

## Abstract

**Objective:**

The infection risk in patients receiving ibrutinib, idelalisib or venetoclax for chronic lymphocytic leukaemia (CLL) or B‐cell lymphoma treated outside of clinical trials is incompletely defined. We sought to identify the severe infection rate and associated risk factors in a ‘real‐world’ cohort.

**Methods:**

We conducted a retrospective cohort study of adult patients with CLL or lymphoma treated with ibrutinib, idelalisib or venetoclax.

**Results:**

Of 67 patients identified (ibrutinib *n* = 53, idelalisib *n* = 8 and venetoclax *n* = 6), 32 (48%) experienced severe infection. Severe infection occurred at a rate of 65 infections per 100 person‐years, with a median of 17.8 months of therapy. Median time to first infection (IQR) was 5.4 months (1.4–15.9). Poor baseline Eastern Cooperative Oncology Group (ECOG) performance status and high Charlson Comorbidity Index (CCI) score associated with increased risk of severe infection [hazard ratios (95% CI) 1.57 (1.07–2.31, *p* = .018) and 1.3 (1.05–1.62, *p* = .016) respectively].

**Conclusion:**

The severe infection rate for patients receiving ibrutinib, idelalisib or venetoclax for lymphoma and CLL exceeded those reported in clinical trials. Patients with poor ECOG or high CCI should be closely monitored for early signs of infection and prevention strategies actively pursued. Further prospective research is required to define optimal antimicrobial prophylaxis recommendations.

**Novelty Statements**

**What is the new aspect of your work?**

Patients receiving ibrutinib, idelalisib or venetoclax for mature B‐cell leukaemia/lymphoma in a ‘real‐world’ setting are at high risk of infection, requiring hospitalisation and/or intravenous antibiotic therapy.

**What is the central finding of your work?**

Poor performance status and a higher comorbidity burden associates with an increased risk of infection in these patients.

**What is the specific clinical relevance of your work?**

Patients receiving these therapies should be monitored with a high index of suspicion for infection and appropriate infection prevention strategies employed.

## 1 INTRODUCTION

Oral novel therapies are increasingly utilised in the treatment of haematological malignancies. Approved agents used in mature B‐cell leukaemia/lymphoma include ibrutinib, idelalisib and venetoclax, first in class inhibitors of Bruton's Tyrosine Kinase (BTK), phosphatidylinositol 3 kinase (PI3K) and B‐cell lymphoma 2 (BCL2) proteins respectively. These therapies are approved in Australia for the treatment of chronic lymphocytic leukaemia (CLL) and small lymphocytic lymphoma (SLL). Additional registered indications for lymphoma include Waldenstrom macroglobulinemia (WM) and mantle cell lymphoma (MCL) (ibrutinib), and follicular lymphoma (FL) (idelalisib).

These targeted agents have established themselves as effective non‐chemotherapy therapeutics that are generally well tolerated. There is, however, an increasing appreciation of an associated infection risk.^1^, ^2^, ^3^ The risk of infection should be considered in the context of immune dysfunction resulting from the underlying haematological malignancy, which is a hallmark of CLL.^4^ Factors associated with immune dysfunction in CLL include reduced T‐cell and natural killer cell function, defective antibody dependent cellular cytotoxicity and neutrophil function and reduced complement activity.^2^, ^4^ Hypogammaglobulinemia is variably associated with an increased risk of infection.^4^ The exposure adjusted rate of major infection in treatment naïve CLL patients has been reported to be 8.5 per 100 person‐years.^5^ Patient‐specific risk factors for infection include age, comorbidities, functional status, number and type of prior lines of therapy, disease stage and refractoriness to treatment, and type of therapy.^2^

In clinical trials using ibrutinib alone or in combination with a monoclonal antibody, the rate of severe infection ranged from 12.8% to 45%.^6^, ^7^, ^8^, ^9^, ^10^ Median follow‐up ranged from 27 to 65 months, with varying patient demographics. A systematic review of 22 prospective clinical trials using ibrutinib for a range of haematological malignancies, reported an overall severe infection rate of 28%.^11^ ‘Real world’ data for ibrutinib are limited to a few retrospective cohort studies, with heterogeneous reporting of infectious outcomes. Severe infection rates ranged from 2% to 25% with median follow‐up from 12 to 19.7 months in patients being treated for CLL or lymphoma.^5^, ^12^, ^13^, ^14^, ^15^ One study reported the incidence rate of severe infection to be 37.5 per 100 person‐years.^5^

The increased risks of *Pneumocystis jiroveci* pneumonia (PJP) and cytomegalovirus (CMV) infections are well established with idelalisib, with safety communications mandating PJP antimicrobial prophylaxis and CMV laboratory and clinical monitoring. Clinical trials have reported severe infection rates of 24%–39% (median follow‐up 14 months).^4^, ^16^, ^17^ A ‘real‐world’ study identified that 48.3% of patients with CLL experienced severe infection at a rate of 80.1 events per 100 person‐years.^18^ In contrast, in clinical trials using venetoclax for previously untreated and relapsed/refractory (RR) CLL, severe infections occurred in 17.5%–22% of patients (median follow‐up 16–28.1 months).^19^, ^20^, ^21^ One ‘real‐world’ study on compassionate access venetoclax reported a severe infection rate of 25.4%.^22^

In summary, reported infection rates in the ‘real‐world’ setting for these novel therapies remains limited, despite increasing use in clinical practice. We sought to define rates of severe infection in the context of ibrutinib, idelalisib or venetoclax treatment for lymphoma and chronic lymphocytic leukaemia, and to identify associated risk factors.

## 2 METHODS

### 2.1 Study design

Single centre, retrospective cohort study across five sites of a tertiary‐level hospital network serving a patient population catchment of 1.5 million in the Australian state of Victoria. Following institutional human research ethics committee approval, pharmacy electronic dispensing records were filtered to identify all patients who had received ibrutinib, idelalisib or venetoclax since October 2014 until June 2021 (which predates widespread community transmission of Sars‐Cov‐2 in Victoria).^23^ Electronic medical records were reviewed for patient demographic data, baseline Eastern Cooperative Oncology Group (ECOG) performance status and Charlson Comorbidity Index (CCI) score, treatment exposure and history and baseline antimicrobial prophylaxis usage. Electronic pathology records were accessed to define baseline serum immunoglobulin G (IgG) levels, serological findings for hepatitis B, human immunodeficiency virus (HIV) and cytomegalovirus (CMV), and evidence of latent tuberculosis (TB) infection.

The pre‐specified primary outcome of our analysis was rate of severe infection, defined as grade 3 or higher, according to the National Cancer Institute Common Terminology Criteria for Adverse Events (CTCAE), version 5.^24^ Secondary outcomes were all‐cause and infection related mortality (IRM). Subjects were followed for the primary outcome up to 30 days from end of treatment. Secondary outcomes were followed for up to 30 days from the end of treatment or 30 days from the onset of infective symptoms, whichever was later.

Severe infection episodes were identified by review of hospital discharge and inpatient documentation. Data specific to each infection episode were collected, including infection onset setting (community, hospital, residential care facility), CTCAE severity grade, infection site and source, microbiological findings, absolute neutrophil count (ANC), IgG serum level, disease response and hospital length of stay (LOS).

### 2.2 Study population

Adult patients (>18 years) were included if they received one or more month of ibrutinib, idelalisib or venetoclax for the treatment of mature B‐cell leukaemia/lymphoma including CLL/SLL, MCL, WM, FL or Marginal Zone Lymphoma (MZL). Exclusion criteria included diffuse large B‐cell lymphoma, cessation of therapy within 1 month of treatment initiation, treatment for other indications or primary delivery of care at other institutions.

### 2.3 Definitions

The World Health Organisation classification (2016) was used to define CLL/SLL, MCL, WM, FL and MZL.^25^ The Lugano classification was used to stage SLL, MCL, FL and MZL and the Binet classification was used to stage CLL.^26^, ^27^ Disease response at the time of infection was defined as that documented in the most recent medical review. Serum IgG levels were recorded if sampled within 6 months of treatment commencement (baseline) or the onset of infective symptoms (time of infection). Hypogammaglobulinemia was defined as an IgG serum level less than 7.5 g/L. Prior CMV exposure was determined by CMV IgG positivity on serological testing. CMV status was defined as indeterminate if intravenous Ig infusion was administered within 3 months of a positive serology finding.

### 2.4 Statistical analysis

Descriptive statistics were calculated using Microsoft Excel Office 16® and GraphPad Prism 9®. Continuous variables are presented as median with the first and third quartile, or minimum and maximum. Categorical variables are presented as numbers and proportions and were compared using Pearson's chi‐square tests or Fisher's exact tests. Incidence rates and 95% confidence intervals (95% CI) were calculated separately for episode of first severe infection and for all severe infections, per 100 person‐years. Person‐years were calculated from the date of treatment commencement to the date of first severe infection, or the date of last follow‐up during treatment, respectively. The Kaplan–Meier method was used to plot overall survival as a function of time and comparisons between curves were made using the log‐rank test. Univariate analysis for time to first infection was performed using Cox proportional hazards regression with results reported as hazard ratios (HR) and 95% CI. A two‐sided *p* value of .05 was chosen to indicate statistical significance. All statistical analyses were performed using SAS software version 9·4 (SAS Institute, Cary, NC, USA).

## 3 RESULTS

### 3.1 Patient characteristics and treatment data

**FIGURE 1:**
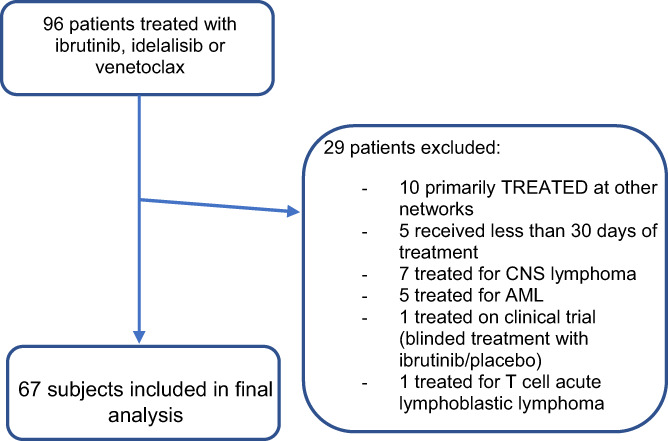
Consort diagram indicating selection of participants included in final analyses.

A total of 96 patients received ibrutinib *or* idelalisib *or* venetoclax from October 2014 to June 2021, of whom 67 were included for analysis (ibrutinib *n* = 53, idelalisib *n* = 8 and venetoclax *n* = 6) (Figure 1). Treatment was delivered as combination therapy for 11 patients, including idelalisib with bendamustine and rituximab (*n* = 1) or rituximab (*n* = 4), and venetoclax with rituximab (*n* = 4) or obinutuzumab (*n* = 2). Median age was 73 years (range 40–93) and 49 (73%) patients were male. Median follow‐up time from treatment commencement to cessation or data collection was 23.3, 4.8 and 3.5 months for ibrutinib, idelalisib and venetoclax, respectively. Of all patients, 29 (43%) were continuing treatment at the time of data collection. Twenty patients (30%) had a baseline Eastern Cooperative Oncology Group (ECOG) performance status of 2–4 and the median Charlson Comorbidity Index (CCI) score of all subjects was 6 (range 3‐10). The most common indications for treatment were CLL/SLL (*n* = 39, 58%) and MCL (*n* = 17, 25%). The majority had RR disease (*n* = 57; 85%) and hypogammaglobulinemia (64% of evaluable patients) at treatment commencement (Table 1).

**TABLE 1:** Baseline demographics and risk factors for infection.

Patient characteristics	All (*n* = 67)	Ibrutinib (*n* = 53)	Idelalisib (*n* = 8)	Venetoclax (*n* = 6)
Age (median, range)	73 (40–93)	74 (40–93)	70 (58–80)	76 (59–82)
Male (*n*, %)	49 (73.1)	42 (79.3)	4 (50)	3 (50)
ECOG				
0–1 (*n*, %)	47 (70.1)	38 (71.7)	6 (75)	3 (50)
2–4 (*n*, %)	20 (29.9)	15 (28.3)	2 (25)	3 (50)
CCI (median, range)	6 (3–10)	5 (3–10)	5.5 (5–7)	7 (3–10)
Lymphoma subgroup (*n*, %)				
CLL/SLL	39 (58.2)	29 (54.7)	4 (50)	6 (100)
MCL	17 (25.4)	17 (32.1)	0	0
WM	7 (10.4)	6 (11.3)	1 (12.5)	0
FL	3 (4.5)	0	3 (37.5)	0
MZL	1 (1.5)	1 (1.9)	0	0
Stage (*n*, %)^a^				
MCL, FL, SLL, MZL				
1–2	4 (6)	3 (5.6)	1 (12.5)	0
3–4	17 (25)	15 (28.3)	2 (25)	0
CLL				
A	2 (3)	0	1 (12.5)	1 (16.7)
B	8 (11.9)	8 (15.1)	0	0
C	28 (41.8)	20 (37.7)	3 (37.5)	5 (83.3)
Relapsed/refractory (*n*, %)	57 (85.1)	46 (86.8)	8 (100)	3 (50)
Hypogammaglobulinemia (*n*, %)^b^	30/47 (63.8)	25/39 (64.1)	1/3 (33)	4/5 (80)
Risk factors				
Prior T cell depleting agent^c^	3 (4)	2 (4)	0	1 (17)
Prior stem cell transplant (SCT)^d^	6 (9)	5 (9)	1 (13)	0
Prolonged corticosteroid use^e^	4 (6)	3 (6)	0	1 (17)
Structural lung disease	13 (19)	9 (17)	3 (38)	1 (17)
Chronic inflammatory disease	7 (10)	6 (11)	0	1 (17)
Duration of treatment (months) (median, interquartile range)	17.8 (5.8–36.4)	23.3 (8.4–38.6)	4.8 (1.8–17.7)	3.5 (2.4–9.0)
Number of prior therapies (median, range)	1 (0–6)	1 (0–6)	1.5 (1–5)	0.5 (0–5)

Abbreviations: CCI, Charlson Comorbidity Index; CLL, chronic lymphocytic leukemia; ECOG, Eastern Cooperative Oncology Group; FL, follicular lymphoma; MCL, mantle cell lymphoma; MZL, marginal zone lymphoma; SLL, small lymphocytic lymphoma; WM, Waldenstrom macroglobulinaemia.

^a^ Not applicable for WM *n* = 7, not documented *n* = 1 (MCL).

^b^ No data available for 20 patients.

^c^ Purine analogue or alemtuzumab use within 12 months.

^d^ Autologous = 5, allogeneic = 1 (ibrutinib; 18 years prior, for CLL).

^e^ Over 20 mg per day of prednisolone equivalent corticosteroid for 4 weeks or longer.

Antimicrobials were prescribed for PJP prophylaxis in 26 patients (39%), Herpes Simplex Virus (HSV)/Varicella Zoster Virus (VZV) prophylaxis in 27 patients (40%) and mould‐active fungal prophylaxis in one (1%). Baseline serology review identified six patients with latent hepatitis B (data not available for 17), no patients with latent TB (data not available for 39), one HIV positive patient (undetectable viral load; data not available for 19) and 21 patients with CMV IgG positivity (data not available for 32; three patients receiving idelalisib).

### 3.2 Infection related outcomes and risk factors

**FIGURE 2:**
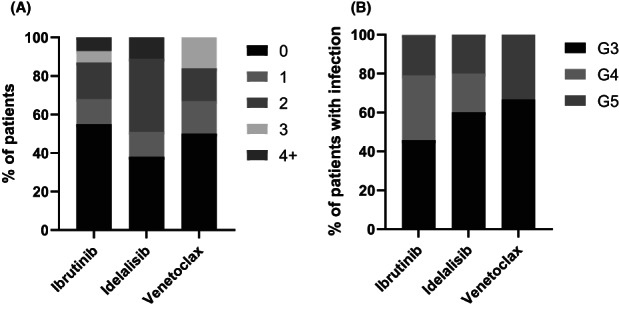
(A) Number of severe infections per subject. (B) Highest CTCAE infection grade per patient with infection. CTCAE, Common Terminology Criteria for Adverse Events; G3, grade 3; G4, grade 4; G5, grade 5.

Thirty‐two (48%) of the 67 patients experienced at least one severe infective episode (Table 2). The number of patients with at least one severe infection during ibrutinib, idelalisib or venetoclax treatment was 24 (45%), 5 (63%), and 3 (50%) respectively. Most subjects with severe infection experienced multiple infection episodes with one severe infection occurring in nine patients (28%), two infections in 14 patients (43.8%), three infections in four patients (12.5%) and four or more infections in five patients (15.6%) (Figure 2A). A total of 79 severe infection episodes were recorded with a median of two hospitalisations (range 1‐8) per patient. The associated median (IQR) cumulative length of stay was 20.5 (8–39.3) days. The incidence rate for first severe infection was 39 per 100 person‐years. The incidence rate for all severe infection episodes was 65 per 100 person‐years (Table 3).

**TABLE 2:** Outcomes for infection and mortality.

	All (*n* = 67)	Ibrutinib (*n* = 53)	Idelalisib (*n* = 8)	Venetoclax (*n* = 6)
Proportion of patients with severe infection				
Patients with 1+ severe infection (*n*, %)	32 (47.7)	24 (45.3)	5 (62.5)	3 (50)
Recurrent infections				
Number of severe infections per patient (median, range)	2 (1–9)	2 (1–9)	2 (1–4)	2 (1–3)
Time to infection				
Time to first infection (median [months], interquartile range)	5.4 (1.4–15.9)	7.6 (1.5–17.9)	1.4 (1.3–3.0)	1.4 (0.9–2.0)
Hospitalisation and mortality data				
Number of hospitalisations per patient (median, range)	2 (1–8)	2 (1–8)	1 (1–3)	1 (1–2)
Total days of hospitalisation per patient (median, interquartile range)	20.5 (8–39.3)	21 (6.75–43.75)	28 (15.5–33.5)	8 (6–11)
All‐cause mortality (*n*, %)	10 (14.9)	7 (13.2)	1 (12.5)	2 (33.3)
Infection‐related mortality (*n*, %)	7 (10.4)	5 (9.4)	1 (12.5)	1 (16.7)

**TABLE 3:** Incidence rate of severe infection.

	Number of infections	Person‐years of follow‐up	Incidence rate of severe infection per 100 person‐years (95% CI)
First severe infection			
All	32	81.3	39 (27–56)
Ibrutinib	24	73.5	33 (21–49)
Idelalisib	5	4.8	104 (34–243)
Venetoclax	3	3.0	100 (20–292)
All severe infections			
All	79	121.5	65 (51–81)
Ibrutinib	62	110	56 (43–72)
Idelalisib	11	7.5	147 (73–262)
Venetoclax	6	3.9	154 (56–335)

Abbreviation: CI, confidence interval.

Fifty infections (63.3%) were CTCAE grade 3 and 18 (22.8%) were grade 4. Seven patients (22% of those with severe infection) died of infection; the highest‐grade of infection that occurred in each patient with infection is represented in Figure 2B. The median time to first infection (IQR) was 5.4 months (1.4–15.9) and most infections occurred within the first 18 months (55 infections, 69.6%) (Supplemental Table 1). The following disease response assessments were documented prior to each infection episode: complete response (*n* = 27; 34.2%), partial response (*n* = 32; 40.5%), not yet assessed (*n* = 16; 20.3%), no response (*n* = 3; 3.8%) and relapsed disease (*n* = 1; 1.3%). Hypogammaglobulinemia was present at the time of 36 (45.6%) infections with a median IgG serum level (range) of 4.1 (0.5–7 g/L; unknown IgG level for 33 episodes of infection). Six (7.6%) infections were associated with neutropenia (ANC <1 × 10^9^/L) within 48 h of infection onset.

Over the 7‐year study period, 10 (14.9%) of the 67 patients died. The median time from commencement of treatment to death was 5.7 months (IQR 2.2–10.9 months). The cause of death was progressive haematological malignancy in three (4.4%) and infection in seven (10.4%). Eight patients (25%) who experienced severe infection died within the follow‐up period compared to two (5.7%) who did not experience severe infection. There was no difference in overall survival between patients with severe infection and those without (log‐rank *p* = .14; Supplemental Figure 1).

Poor performance status (ECOG) and a high CCI score were associated with an increased risk of severe infection with hazard ratios of 1.57 (95% CI 1.07–2.31, *p* = .018) and 1.3 (95% CI 1.05–1.62, *p* = .016) respectively (Table 4). Twenty‐nine patients (51%) with RR disease had a severe infection compared to three (30%) of those with previously untreated disease, HR 1.44 (95% CI 0.42–4.88, *p* = .553).

**TABLE 4:** Univariate Cox regression analysis of risk factors for severe infection.

Variable	HR	(95% CI)	*p* value
Age	1.03	(0.99–1.07)	.143
Male gender	0.61	(0.29–1.29)	.190
ECOG	1.57	(1.07–2.31)	.018
Charlson Comorbidity Index	1.30	(1.05–1.62)	.016
Number of prior therapy lines	1.25	(0.94–1.64)	.112
Relapsed/refractory disease	1.44	(0.42–4.88)	.553
Baseline hypogammaglobulinemia	1.32	(0.53–3.31)	.541
Prior T cell depleting therapy	0.52	(0.07–4.88)	.526
Prior stem cell transplant	0.62	(0.14–2.71)	.522
Prolonged corticosteroid use	1.74	(0.40–7.61)	.450
Structural lung disease	1.82	(0.82–4.03)	.131
Chronic inflammatory disease	0.87	(0.26–2.93)	.813

Abbreviations: CI, confidence interval; ECOG, Eastern Cooperative Oncology Group; HR, hazard ratio.

Of 79 severe infections, 36 (45.6%) were microbiologically proven, 36 (45.6%) clinically defined, five (6.3%) were fever without focus and two (2.5%) were treated as infection without fever. Of the microbiologically proven infections, 17 (47.2%) were bacterial, 17 (47.2%) viral and two (5.6%) fungal; 10 (27.8%) of these infections were caused by opportunistic pathogens, occurring in seven (10.4%) patients (Supplemental Table 2). Invasive fungal infections (IFI) occurred on two occasions (both fatal): *Candida glabrata* sepsis during ibrutinib treatment and *Lomentospora prolificans* sepsis while on idelalisib. No PJP infections were observed. The patients who experienced HSV infection (*n* = 2) or VZV reactivation (*n* = 1) (Supplemental Table 2) had not received prophylaxis. All patients with latent hepatitis B (*n* = 6) received concomitant lamivudine or entecavir and viral reactivation was not detected during follow‐up. The HIV‐infected patient did not experience any severe infections. One patient experienced CMV viraemia (reactivation) while receiving idelalisib. Two episodes of CMV infection occurred in a patient with unknown CMV baseline serology receiving venetoclax and a patient with indeterminate CMV baseline serology receiving idelalisib. There were no Sars‐Cov‐2 infections.

There were 43 (54%) episodes of respiratory infection and 16 (20%) episodes of sepsis (Supplemental Table 3). Of the 79 cases of severe infection, 63 (80%) were community acquired, eight (10%) were acquired in residential care facilities and eight (10%) had onset in the hospital setting. Of the community and residential care acquired infections, the median time between symptom onset and hospital admission was two days (IQR 1‐6).

## 4 DISCUSSION

This study demonstrated a high rate of severe infection in a ‘real‐world’ cohort of patients with long‐term follow‐up. The severe infection rate was higher than that reported in clinical trials and prior post‐marketing studies, with one in 10 patients dying from infection‐related causes. Over two‐thirds of those with severe infection experienced recurrent events. Our report details the proportion of patients affected by severe infection and provides additional data on the exposure‐adjusted incident rate. The incidence rate for all infective episodes was 1.5 and 1.8 times higher than those reported in prior ‘real world’ studies for ibrutinib and idelalisib respectively.^5^, ^18^ Our analysis is weighted towards ibrutinib and the treatment of CLL, for which data is largely presented separately. There is, however, descriptive value in our small case series of idelalisib and venetoclax given only isolated case reports and single ‘real‐world’ case series exist in the literature. The period of observation in this study was shorter than of prospectively reported clinical trials, yet a high rate of outcome occurrence was captured. It is noteworthy that this higher than anticipated infection rate was detected in a retrospective analysis where some infective events may have been missed.

Our study population was generally older than those reported in clinical trials.^4^, ^6^, ^7^, ^8^, ^9^, ^10^, ^16^, ^17^, ^19^, ^20^, ^21^ A high proportion demonstrated baseline hypogammaglobulinemia and most were treated for RR disease. One‐third of all subjects had an ECOG performance status between 2 and 4, representing those who are typically excluded from clinical trial and may be under‐represented in other ‘real‐world’ studies. We report that poor ECOG performance status and a high CCI score is associated with an increased risk of severe infection. Neutropenia, RR disease, and number of prior lines of therapy have previously been associated with an increased infection risk. In contrast, partial immune reconstitution with increased IgA levels after ibrutinib commencement has been associated with a reduced risk of infection.^5^, ^8^, ^12^ Our study was likely underpowered to detect a significant impact of these risk factors, did not assess IgA levels and had insufficient data to analyse the impact of hypogammaglobulinemia. Most infections occurred within the first 18 months, which aligns with other studies reporting the majority of infections occurring early in treatment, within the first 6–12 months^8^, ^12^ or waning over time.^6^ This may be explained from partial reconstitution of humoral immunity^8^, ^15^ or increasing depth of disease response over time.^9^ Given the high rates of severe infection observed, we recommend consideration of appropriate infection prevention strategies. Antimicrobial prophylaxis was prescribed for nearly half of all subjects which may have mitigated infection rates. No patient experienced hepatitis B reactivation in the context of appropriate viral suppressive therapy. Further research into directed antimicrobial prophylaxis for carefully selected patients is required.

Our study was limited also by a scarcity in the documentation of vaccination history and related laboratory assessment of vaccination responses. The effectiveness of vaccination in the prevention of severe infections (e.g., influenza, invasive pneumococcal disease) is an important consideration which is particularly topical given the current Sars‐CoV‐2 pandemic, which largely post‐dated our analysis in the Australian context. Serological responses to influenza, hepatitis B and Sars‐CoV‐2 vaccines are reduced in patients receiving ibrutinib^28^, ^29^ and ibrutinib, idelalisib and venetoclax are scheduled as indefinite or long‐term therapies. Pre‐treatment vaccination is ideal, where possible, but should be considered nonetheless once treatment has commenced. Many of the infections experienced by our subjects were non‐opportunistic and not directly vaccine preventable. However, patient education to recognise early signs of infection may allow for earlier presentation for treatment.

The treatment setting continues to evolve, with increased access to ibrutinib and venetoclax in the first line and limited clinical utility of idelalisib due to recognised infection risk. Furthermore, second generation BTK inhibitors including acalabrutinib and zanubrutinib have emerged.^30^ They have increased specificity for the BTK receptor and fewer off target effects, including inhibition of interleukin‐2‐inducible T‐cell kinase, which has a key role in T‐cell maturation and function.^4^, ^11^, ^30^ The risk of severe infection in this population requires ongoing review in the contemporary treatment setting.

## 5 CONCLUSION

Our data demonstrates that the ‘real‐world’ population receiving ibrutinib, idelalisib or venetoclax for chronic lymphocytic leukaemia or lymphoma, particularly those with poor baseline ECOG and high CCI, are at a high risk of severe infection. Patients should be closely monitored for the development of infection and appropriate infection prevention strategies should be employed. Our findings support and amplify safety signals from existing literature however, larger prospective studies are needed to identify sub‐populations at greatest risk.

## AUTHOR CONTRIBUTIONS

Amanda Tey analysed data and wrote manuscript. James Schwarer, Robert Raffa and Emily Shi collected and analysed data. Eldho Paul performed statistical analysis. Claire Dendle and Stephen Opat analysed data. Jake Shortt analysed data and wrote manuscript. All authors reviewed the manuscript prior to submission.

## CONFLICT OF INTEREST STATEMENT

Jake Shortt has received research funding from Astex Pharmaceutical Inc., Amgen and Bristol Myers Squibb/Celgene; Jake Shortt has served on Advisory Boards for Novartis, BMS, Mundipharma and Astellas. Stephen Opat has provided consultancy to AbbVie, Astra Zeneca, Janssen and Roche; Stephen Opat has received research funding from Amgen and Beigene; Stephen Opat has received honoraria from AbbVie, Astra Zeneca, Celgene, CSL Behring, Gilead, Janssen, Merck, Roche and Takeda; Stephen Opat has served on Advisory Boards for AbbVie, Astra Zeneca, Celgene, CSL Behring, Gilead, Janssen, Merck, Roche and Takeda.

## ETHICS STATEMENT

This research was prospectively approved by the Monash Health Human Research Ethics Committee; local reference number: RES‐19‐0000‐558Q.

## Supporting information


**Supplemental Table 1.** Occurrence of severe infection over time. ^†^Latest disease response assessment at time of infection: complete remission *n* = 9, partial remission *n* = 10, relapsed disease *n* = 1.
**Supplemental Table 2.** Classification of microbiologically proven infections. ^†^Opportunistic organisms.
**Supplemental Table 3.** Infection site.
**Supplemental Figure 1.** Overall survival (OS) from commencement of treatment. Median OS no infection not reached vs infection 1767 days; log‐rank *p* = .14.

## Data Availability

The data that support the findings of this study are available from the corresponding author upon reasonable request.
