# Supplementary material for: High risk of infection in ‘real‐world’ patients receiving ibrutinib, idelalisib or venetoclax for mature B‐cell leukaemia/lymphoma
Source: Eur J Haematol. 2023 Feb 15;110(5):540–7. doi: 10.1111/ejh.13928 (PMC10952205; doi:10.1111/ejh.13928)
Supplement: Supplementary file 1 — Supplemental Table 1. Occurrence of severe infection over time. †Latest disease response assessment at time of infection: complete remission n = 9, partial remission n = 10, relapsed disease n = 1. Supplemental Table 2. Classification of microbiologically proven infections. †Opportunistic organisms. Supplemental Table 3. Infection site. Supplemental Figure 1. Overall survival (OS) from commencement of treatment. Median OS no infection not reached vs infection 1767 days; log‐rank p = .14. [file EJH-110-540-s001.docx]

**Supplemental material**

|  | **0-6 months** | **6-12 months** | **12-18 months** | **18-24 months** | **24+ months** |
| --- | --- | --- | --- | --- | --- |
| **Number of severe infections (n, %)** | 31 (39.2) | 14 (17.7) | 10 (12.7) | 4 (5.1) | 20 (25.3) † |
| **Cumulative number of patients with first infection (n, %)** | 16 (24) | 22 (33) | 25 (37) | 26 (39) | 32 (48) |

Supplemental table 1. Occurrence of severe infection over time. † Latest disease response assessment at time of infection: complete remission n=9, partial remission n=10, relapsed disease n=1

Supplemental figure 1: Overall survival (OS) from commencement of treatment. Median OS no infection not reached *vs* infection 1767 days; log-rank p=0.14

| **Organism class** | **Organism (number)** | **Site of isolate (number)** |
| --- | --- | --- |
| **Bacterial** (n=17) | | |
| Gram positive organisms n=7 | *Staphylococcus aureus* (2) | Blood and Wound (1), wound (1) |
|  | *Staphylococcus aureus* and *streptococcus anginosus* (1) | Lung (1) |
|  | *Streptococcus anginosus* (1) | Eye (1) |
|  | *Streptococcus pneumoniae* (1) | Blood (1) |
|  | *Actinomyces oris* (1) | Blood (1) |
|  | *Ruminococcus gnavus* (1) | Blood (1) |
| Gram-negative organisms n=9 | *Escherichia coli* (4) | Blood (2), blood and urine (2) |
|  | *Haemophilus parainfluenzae* (1) | Wound (1) |
|  | *Moraxella catarrhalis* (1) | Lung (1) |
|  | *Raoultella planticola* (1) | Blood (1) |
|  | *Pasteurella canis* (1) | Lung (1) |
|  | *Campylobacter spp*. (1) | Gastrointestinal (GI) (1) |
| Other n=1 | *Mycoplasma pneumoniae* (1)† | Lung (1) |
| **Viral (**n=17) | | |
|  | Herpes Simplex Virus (2)† | Blood (1), wound (1) |
|  | Varicella Zoster Virus (1)† | Wound (1) |
|  | Cytomegalovirus (3)† | Blood (1), blood and GI (2) |
|  | Epstein Barr virus (1)† | Blood (1) |
|  | Rhinovirus/Enterovirus (7) | Ear, nose and throat (ENT) (7) |
|  | Picornavirus (1) | ENT (1) |
|  | Parainfluenza 3 (1) | ENT (1) |
|  | Influenza A (1) | ENT (1) |
| **Fungal (**n=2) | | |
|  | *Candida glabrata* (1)† | Blood (1) |
|  | *Lomentospora prolificans* (1)† | Blood (1) |

Supplemental table 2. Classification of microbiologically proven infections. † opportunistic organisms

| **Site of infection** | **Number of infections** | **Percentage of all infections** |
| --- | --- | --- |
| Respiratory | 43 | 54% |
| Blood | 16 | 20% |
| Unknown | 7 | 9% |
| Skin/wound | 6 | 8% |
| Abdominal | 5 | 6% |
| Device | 1 | 1% |
| Gastrointestinal | 1 | 1% |

Supplemental table 3. Infection site
